# An Integrated Modeling and Experimental Approach to Study the Influence of Environmental Nutrients on Biofilm Formation of *Pseudomonas aeruginosa*

**DOI:** 10.1155/2015/506782

**Published:** 2015-04-14

**Authors:** Zhaobin Xu, Sabina Islam, Thomas K. Wood, Zuyi Huang

**Affiliations:** ^1^Department of Chemical Engineering, Villanova University, Villanova, PA 19444, USA; ^2^Departments of Chemical Engineering and Biochemistry and Molecular Biology, Pennsylvania State University, University Park, PA 16802, USA; ^3^The Center for Nonlinear Dynamics & Control (CENDAC), Villanova University, Villanova, PA 19444, USA; ^4^Villanova Center for the Advancement of Sustainability in Engineering (VCASE), Villanova University, Villanova, PA 19444, USA

## Abstract

The availability of nutrient components in the environment was identified as a critical regulator of virulence and biofilm formation in* Pseudomonas aeruginosa*. This work proposes the first systems-biology approach to quantify microbial biofilm formation upon the change of nutrient availability in the environment. Specifically, the change of fluxes of metabolic reactions that were positively associated with* P. aeruginosa* biofilm formation was used to monitor the trend for* P. aeruginosa* to form a biofilm. The uptake rates of nutrient components were changed according to the change of the nutrient availability. We found that adding each of the eleven amino acids (Arg, Tyr, Phe, His, Iso, Orn, Pro, Glu, Leu, Val, and Asp) to minimal medium promoted* P. aeruginosa* biofilm formation. Both modeling and experimental approaches were further developed to quantify* P. aeruginosa* biofilm formation for four different availability levels for each of the three ions that include ferrous ions, sulfate, and phosphate. The developed modeling approach correctly predicted the amount of biofilm formation. By comparing reaction flux change upon the change of nutrient concentrations, metabolic reactions used by* P. aeruginosa* to regulate its biofilm formation are mainly involved in arginine metabolism, glutamate production, magnesium transport, acetate metabolism, and the TCA cycle.

## 1. Introduction 

Forming biofilms is one of the major strategies implemented by pathogens to survive antibiotic treatment, and biofilms cause chronic human infections [1]. It is reported that 10 to 1000 times higher doses of antibiotics are required to treat pathogens in a biofilm [2, 3]. Investigation of the factors that influence microbial biofilm formation is thus important for combating biofilms associated with pathogens. This research topic has attracted extensive experimentation. For example, the genes that are upregulated during* P. aeruginosa* biofilm formation have been identified experimentally [4]. Since microbial metabolism depends on the interaction of hundreds to thousands of metabolic reactions, systems-level modeling approaches that can incorporate existing experimental data are essential to pinpoint the factors that play a crucial role in microbial biofilm formation.

Extensive research on modeling microbial biofilms has been conducted in the last 30 years (see Wang and Zhang, 2010 [5], for a detailed review). However, all these models use the Monod kinetics to quantify the microbial growth rate, that is, the growth rate *μ* = *V*_*m*_ *C*_*s*_/(*K*_*m*_ + *C*_*s*_) in which *C*_*s*_ is the substrate concentration and *V*_*m*_ and *K*_*m*_ are the maximum growth rate and half-velocity constant, respectively. No information is thus obtained from these models to quantify the influence from the environmental nutrients on the intracellular microbial metabolism and thus the biofilm formation. Moreover, none of these methods has systematically studied biofilm formation based on the availability of environmental nutrients. On the other hand, genome-scale models have been shown to integrate the extracellular environmental conditions with the intracellular metabolic reactions [6]. On the basis of the genome-scale model developed by Oberhardt et al., 2008 [7], the Ines Thiele group from University of Iceland [8] proposed the first systems-biology approach to identify gene targets to terminate the growth of* P. aeruginosa* in the biofilm. This approach mainly constrained the nutrient uptake rates to mimic the mature biofilm environment and performed single/double* in silico* gene knockouts to determine the gene targets essential for microbial growth in the biofilm. However, biofilm formation ability of the mutants, which may not be reflected by the microbial growth, has not been studied in this approach. In particular, a slowly growing mutant may form more biofilm than a fast-growing mutant. To address this problem, we developed a systems-level approach to identify gene targets to prevent the biofilm formation of* P. aeruginosa*, and we found that genes essential to microbial growth are not necessarily good targets to treat* P. aeruginosa* biofilm, as most of the essential gene mutants form more biofilms before they are completely eliminated by antibiotics [9]. While these existing metabolic modeling approaches mainly focus on the influence of metabolic genes on biofilm formation, no modeling approach has been published to quantify the microbial biofilm formation upon the change in the environmental nutrient conditions; however, the availability of environmental nutrients has been shown to influence microbial biofilm formation. For example, some strains of* Escherichia coli* K-12 and* Vibrio cholerae* were reported not to form biofilms in minimal medium unless the medium is supplemented with amino acids [10]. In addition to amino acids, ions such as ferrous ions, phosphate, and sulfate also play an important role in regulating biofilm formation [11, 12]. For example, phosphate influences the biofilm formation of* Pseudomonas fluorescens* by controlling secretion of adhesion LapA to the surface [13], and ferrous ions are important for the maturation of* P. aeruginosa* biofilms [14].

In this work, we developed the first systems-biology approach to quantify* P. aeruginosa* biofilm formation based upon the availability of environmental nutrients that include 20 amino acids and three ions (i.e., sulfate, phosphate, and ferrous ions). Specifically, we further extended our previous modeling approach that was developed to determine gene targets to eliminate* P. aeruginosa* before it forms a biofilm [9]. The idea behind that approach was using the change of fluxes of reactions positively associated with* P. aeruginosa* biofilm formation (henceforth biofilm-associated reactions) to classify single mutants into different categories. Based upon these categories, targets to prevent* P. aeruginosa* biofilm formation were identified. Unlike our previous modeling approach, the availability of a specific nutrient component, instead of the expression of a specific metabolic gene, was manipulated here. In addition, a new formula was defined to quantify the microbial biofilm formation from the flux change through the biofilm-associated reactions. The modeling work was based upon the metabolic model of* P. aeruginosa* originally developed and updated by the Papin group at the University of Virginia (Oberhardt et al., 2008 and 2010 [7, 15]). In particular, the reaction “Rha-(a1,3)-GlcNac-pyrophosphorylundecaprenol synthesis” (RHA1GLCNACPPUNDs) was altered in the updated model to produce guanosine monophosphate (GMP) instead of uridine monophosphate (UMP). The updated model (i.e., Oberhardt et al., 2010 [15]) was obtained from Papin group by personal communication. This model was used because its previous version has been recently used to successfully predict the phenotype of* P. aeruginosa* in biofilms (refer to Sigurdsson et al., 2012 [8], and Xu et al., 2013 [9], for two examples). Based on the selected metabolic model, flux changes through reactions that were identified to be positively associated with* P. aeruginosa* biofilm formation (i.e., biofilm-associated reactions) were used to quantify the biofilm formation of* P. aeruginosa* upon the change in the availability of nutrient components including 20 amino acids and three ions (i.e., ferrous ions, phosphate, and sulfate). These nutrient components were selected in this work due to their importance for the regulation of* P. aeruginosa* biofilm formation. Data from the literature were used to validate our results for the 20 amino acids. In addition, we conducted the first complete experimental evaluation of* Pseudomonas aeruginosa* biofilm formation for a large change in the concentrations of sulfate, phosphate, and ferrous ions to verify the results for these three ions. Both starved and rich nutrient conditions for each nutrient component were studied in this work.

## 2. Results

**Figure 1:**
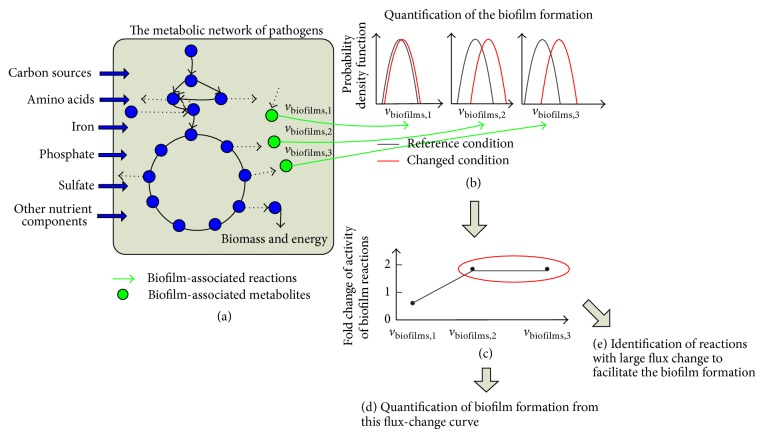
An illustrative example to show the proposed approach for quantifying biofilm formation of* P. aeruginosa* for different availabilities of a specific nutrient component. The availability of the target nutrient component is represented by its maximum uptake rate in the flux balance analysis. A large uptake rate is used for the abundant nutrient condition, while a small uptake rate is assigned for the starving nutrient (a). The fluxes of those biofilm-associated reactions are then sampled for both reference and changed nutrient conditions and represented as flux distributions (b). The horizontal axe represents the metabolic fluxes through the *i*th biofilm-associated reactions, that is, *v*_biofilm,*i*_, while the vertical axe represents the occurrence probability of a specific metabolic flux. Upon the change of the nutrient availability from the reference to changed condition, the change of fluxes through each biofilm-associated reaction is quantified, which is then used to obtain the flux-change curve over all biofilm-associated reactions (c). Finally, the flux change curve is used to quantify biofilm formation capability of* P. aeruginosa* (d). In particular, a large flux increase in most biofilm-associated reactions indicates a high trend to form more* P. aeruginosa* biofilm. The metabolic reactions with large flux change during the biofilm formation were further identified, as they may imply the mechanisms used by* P. aeruginosa* to form a biofilm (e).

An illustrative example is shown in Figure 1 to outline our approach to quantify the formation of* P. aeruginosa* biofilm for different availabilities of nutrient components in the environment. This is followed by a detailed description of the results for* P. aeruginosa* biofilm formation upon the change in the availability of each of 20 amino acids, ferrous ions, phosphate, and sulfate.

### 2.1. An Illustration Example of the Proposed Approach

Figure 1 shows an illustrative example to outline our approach for quantifying biofilm formation ability of* P. aeruginosa* for a specific nutrient condition. First, the change of the nutrient availability can be mimicked by changing the maximum uptake rate of the nutrient component in the flux balance analysis [17] (refer to Section 4 for more details of flux balance analysis). For example, a zero maximum uptake rate is assigned to arginine for the minimal medium without arginine (referred to as reference nutrient condition), and a maximum 10 mmol gDW^−1^ h^−1^ uptake rate, suggested by Oberhardt et al., 2008 [7], is used to mimic the adding of arginine into minimal medium (referred to as the changed nutrient condition). The flux balance analysis is then performed based on the metabolic model and the uptake constraints for both reference and changed nutrient conditions (Figure 1(a)), and the fluxes through the biofilm-associated reactions are sampled and represented as flux distributions for both conditions (Figure 1(b)). Biofilm-associated reactions are 39 reactions that were identified to be positively associated with* P. aeruginosa* biofilm formation from the genetic determinant data published in Müsken et al., 2010 [4]. They are mainly related to nitrite consumption, acetate production, the TCA cycle, carbon dioxide production, pyrimidine metabolism, and oxidative phosphorylation. Interested readers can refer to our previous work [9] for the details of these 39 biofilm-associated reactions. On the basis of the flux distributions shown in Figure 1(b), we further quantified the flux change through each biofilm-associated reaction and obtain a flux-change curve across all biofilm-associated reactions (Figure 1(c)). A criterion named biofilm formation capability is determined from this flux-change curve to quantify the trend for* P. aeruginosa* to form biofilms upon the change of the availability of a specific nutrient component (e.g., adding arginine into minimal medium) (Figure 1(d)). Biofilm formation capability is actually defined as the ratio of the fluxes through biofilm-associated reactions for the changed nutrient condition (e.g., minimal medium with the addition of arginine) over the ones for the reference nutrient condition (e.g., minimal medium without arginine). In particular, enhanced fluxes through most biofilm-associated reactions result in a large value of biofilm formation capability, which indicates that the changed nutrient availability induces* P. aeruginosa* to form more biofilm. Only the availability of a nutrient component is changed in Figure 1, although the developed framework can be applied to study the influence of multiple nutrient components on* P. aeruginosa* biofilm formation. Finally, the biofilm-associated reactions with large flux changes are identified from the flux-change curve (Figure 1(e)). These reactions may reveal the mechanisms used by the pathogen to adjust its biofilm formation under different nutrient conditions.

### 2.2. Influence of the Availability of Amino Acids in the Medium on* P. aeruginosa* Biofilm Formation

**Figure 2:**
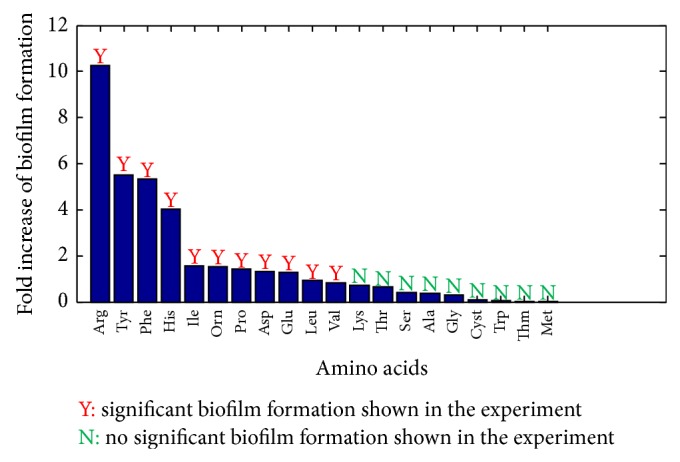
Biofilm formation capability of planktonic* P. aeruginosa* upon adding each of the 20 amino acids into minimal medium. Minimal medium is used as the reference nutrient condition, while adding each of the 20 amino acids is referred to as the changed nutrient condition. The experimental data (Y, N) are obtained from Bernier et al., 2011 [16].

In this section, the trend for planktonic* P. aeruginosa* to form a biofilm upon adding an amino acid to the minimal medium was quantified. The minimal medium was thus used as the reference nutrient condition in which* P. aeruginosa* stayed in the planktonic growth mode. Only one amino acid was added into the minimal medium at a time, which represented a changed nutrient condition. The capability of planktonic* P. aeruginosa* to form a biofilm for each changed nutrient condition was determined and compared with the experimental data presented by Bernier et al., 2011 [16] (Figure 2). A zerofold increase of biofilm formation shown in Figure 2 means the biofilm formation for the changed condition is the same as that for the reference condition. The experiment data show that (1) adding any of the 20 amino acids into the minimum medium results in the biofilm formation; (2) the supplementation of any of the following 11 amino acids, that is, Arg, Tyr, Phe, His, IlE, Orn, Pro, Asp, Glu, Leu, and Val, can significantly enhance biofilm formation; (3) adding any other amino acid only induces minor biofilm formation. As shown in Figure 2, the model predicted biofilm formation matches these observations very well. In particular, the addition of any of the aforementioned 11 amino acids was predicted to enhance at least onefold biofilm formation, while the supplementation of any other amino acids (especially Cyst, Trp, Thm, and Met) only leads to limited biofilm formation.

**Figure 3:**
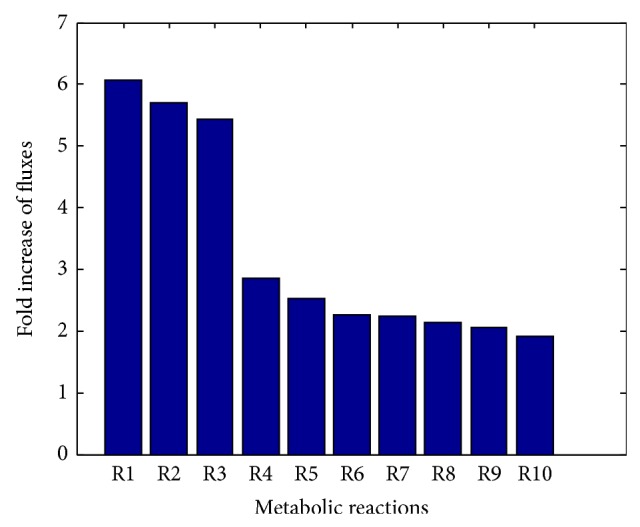
The average flux change of the biofilm-associated reactions. Only the top 10 reactions with the largest average flux change are shown here.

In order to further study the biofilm formation mechanism stimulated by the addition of amino acids, we calculated the flux change through each biofilm-associated reaction upon the addition of each of the aforementioned 11 amino acids that significantly enhance biofilm formation. Based upon this, the average flux change through each biofilm-associated reaction over the addition of the 11 amino acids was calculated. The top 10 biofilm-associated reactions with large average flux change were listed in Table 1, and the average flux change of these reactions was shown in Figure 3. The average flux change through Rxn 1 to Rxn 3 is increased more than 3-fold upon the addition of each of the 11 amino acids into the medium. These three reactions are mainly related to the ammonia production, arginine metabolism, and pyruvate metabolism. On the other hand, Rxn 4 to Rxn 10 have two to threefold increase in their average flux change. These reactions are involved in the arginine metabolism (Rxn 4 and Rxn 7), pyridoxine metabolism (Rxn 5), glutamate production (Rxn 6), pyruvate metabolism (Rxn 8), quorum sensing (Rxn 9), and CO_2_ production (Rxn 10). Therefore, upon the additional availability of amino acids,* P. aeruginosa* form more biofilm mainly through the regulation of arginine metabolism, pyruvate metabolism, and pyridoxine metabolism and the production of ammonia, quorum sensing component, and CO_2_. These observations were implied by some existing literature data. For example, it was reported that the availability of arginine [16] and production of ammonia [18] and CO_2_ [19] are positively correlated with* P. aeruginosa* biofilm formation.

### 2.3. Influence of the Availability of Ferrous Ions, Sulfate, and Phosphate in the Medium on* P. aeruginosa* Biofilm Formation

In the following text, the proposed approach was further applied to quantify* P. aeruginosa* biofilm formation upon different availability levels of inorganic ions that include ferrous ions, sulfate, and phosphate. Experiment for four different concentrations of each ion was performed to quantify the biofilm formation and biomass growth of* P. aeruginosa*. In order to compare the biomass growth rate and biofilm formation across different concentrations of each ion, the biomass growth rate and biofilm formation for one concentration were used as the reference values to normalize the biomass growth rate and biofilm formation for the other three concentrations. The reference concentration for each of the three ions is shown in Table 2. Four concentrations, that is, 0.05-, 0.5-, 1-, and 5-fold, of the reference concentration were investigated experimentally for each ion so that both the starved and rich ion conditions were covered. The details of the experimental design are given in Section 4.

The calculated biomass growth rate and biofilm formation for nonreference concentrations were normalized by the values for the reference concentration. Selecting concentrations other than those shown in Table 2 as the reference concentration will only change the format to present the results but will not change the results. In the calculation, the uptake rate of each target ion was regulated so that the model-predicted biomass growth rate matched the corresponding experimental data. The fluxes through the 39 biofilm-associated reactions were quantified for each ion concentration to quantify the biofilm formation (see Section 4 for the detailed formula). The model predicted biofilm formation capability was then compared to the experimentally measured biofilm formation data.

**Figure 4:**
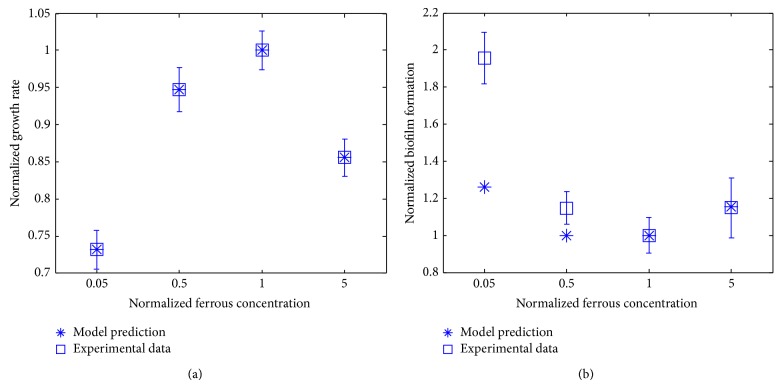
Biomass growth rate (a) and biofilm formation capability (b) for different availabilities of ferrous ions in the medium. The reference ferrous ion concentration is 5 × 10^−4 ^mM.

The biomass growth rate and biofilm formation of* P. aeruginosa* upon the change in the availability of ferrous ions were quantified and compared with our own experimental data (Figure 4). It can be seen from Figure 4(a) that the biomass growth rate increases to its maximum value and then deceases upon the increase of ferrous ions from the starved concentration. On the other hand, the biofilm formation is promoted for a low iron concentration and then repressed for increasing iron concentrations until the iron concentration is high enough. The experimental results shown in Figure 4 are implied by some existing studies that have been undertaken to determine how iron affects biofilm formation by* P. aeruginosa*. For example, researchers found that a minimum amount of free iron was necessary in order for* P. aeruginosa* to form structured biofilm in flow chambers [19, 20] and low iron concentration promoted* P. aeruginosa* biofilm formation in artificial cystic fibrosis sputum medium [21]. In addition, increasing iron concentrations to a certain high value suppressed* P. aeruginosa* biofilm in both microtiter plates and flow chambers [22, 23]. Furthermore, Yang et al. (2007) found that* pqs* quorum-sensing related genes and the level of extracellular DNA (eDNA) were upregulated in low iron conditions promoting* P. aeruginosa* biofilm formation and that increasing iron to high concentrations downregulates* pqs* genes and eDNA formation, thereby decreasing biofilm formation [23]. The experimental result is in agreement with the part that biofilm level is elevated with low iron condition and inhibited with increasing iron to certain concentrations.  Figure 4(b) shows that our modeling approach correctly predicts the trend of the biofilm formation for a variety of iron concentrations. In particular, the predicted biofilm formation for medium and high iron concentration matches experimental data well, especially for the iron concentration equal to 5-fold of the reference concentration. The model prediction also underestimated the biofilm formation for the low iron concentration (i.e., 0.05-fold of the reference iron concentration), which may be due to the lack of the regulation in the model for microbial metabolism which is controlled by quorum sensing signaling (e.g., the* pqs* quorum-sensing mentioned above). Specifically, not all signaling proteins that are regulated by quorum sensing are incorporated in the metabolic network, as they are not the metabolic enzymes that directly regulate bacterial growth.

**Figure 5:**
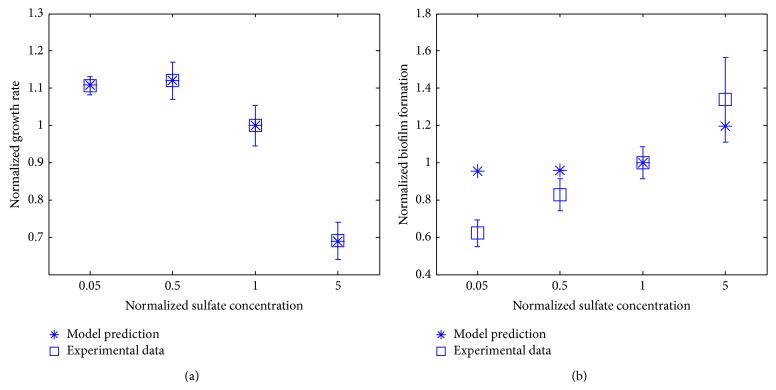
Biomass growth rate (a) and biofilm formation capability (b) for different availabilities of sulfate in the medium. The reference sulfate concentration is 0.05 mM.

The experimental and model-predicted results for the biomass growth rate and biofilm formation upon the change in the availability of sulfate concentrations in the medium are given in Figure 5. In particular, Figure 5(a) shows that the biomass growth rate decreases while the biofilm formation increases upon the increase of the sulfate concentration in the medium. While few existing experimental data were found in the literature on the relationship between sulfate and biofilm formation by* P. aeruginosa*, we here provided new experimental data in this field. Our modeling approach indicates the same trend in* P. aeruginosa* biofilm formation as the one shown in the experimental data. The model-predicted biofilm formation for the medium and high sulfate concentrations matched the measured value very well, while the model overestimated the biofilm formation for the low sulfate concentration.

**Figure 6:**
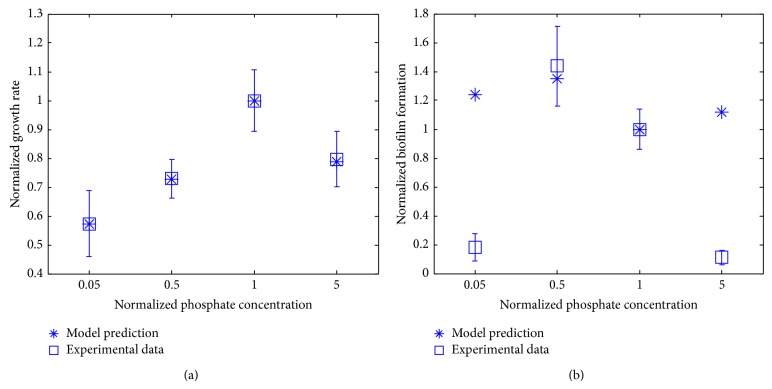
Biomass growth rate (a) and biofilm formation capability (b) for different availabilities of phosphate in the medium. The reference phosphate concentration is represented by 4.93 mM Na_2_HPO_4_ and 2.2 mM KH_2_PO_4_.

The biomass growth rate and the biofilm formation predicted by our modeling approach for these four phosphate concentrations were plotted with the corresponding experimental data in Figure 6. It can be seen that the biomass growth rate increases to its peak value and then decreases upon the increase in the availability of phosphate (Figure 6(a)). On the other hand, the biofilm formation displays the same trend as the biomass growth in the experimental data, as little biofilm is formed for very low or high phosphate concentrations (Figure 6(b)). Low phosphate conditions were shown to trigger virulence in* P. aeruginosa* [24]. A study was conducted where* Caenorhabditis elegans* worms were allowed to feed on nonvirulent* P. aeruginosa* grown in either high or low phosphate medium. Worms that fed on* P. aeruginosa* grown in low phosphate medium developed large red spots on their intestine and 60% of them eventually died (red death syndrome) whereas no nematodes that fed on high phosphate grown* P. aeruginosa* died [24]. This result suggests that there is more biofilm with low phosphate. Further study has elucidated that phosphate depletion leads to increase in biofilm formation as well as expression of PA-I lectin (causes sepsis by disrupting the intestinal epithelial barrier) and pyocyanin (responsible for neutrophil apoptosis) in* P. aeruginosa* [25]. These findings, which were not designed for the starved phosphate conditions, corroborate our experimental result where biofilm formation decreases with increasing phosphate concentration (from 0.5 to 5). Our data provided additional information for the biofilm formation for phosphate starvation condition (0.05-fold of the reference concentration). The model-based predicted biofilm formation by* P. aeruginosa* presents a similar trend to the one shown in the experimental data for low and medium phosphate concentrations. In particular, our modeling approach predicts the biofilm formation for the concentration of 0.5-fold quite well, although it overestimates the biofilm formation for both the low and high phosphate concentrations (i.e., 0.05- and 5-fold, resp.).

**Figure 7:**
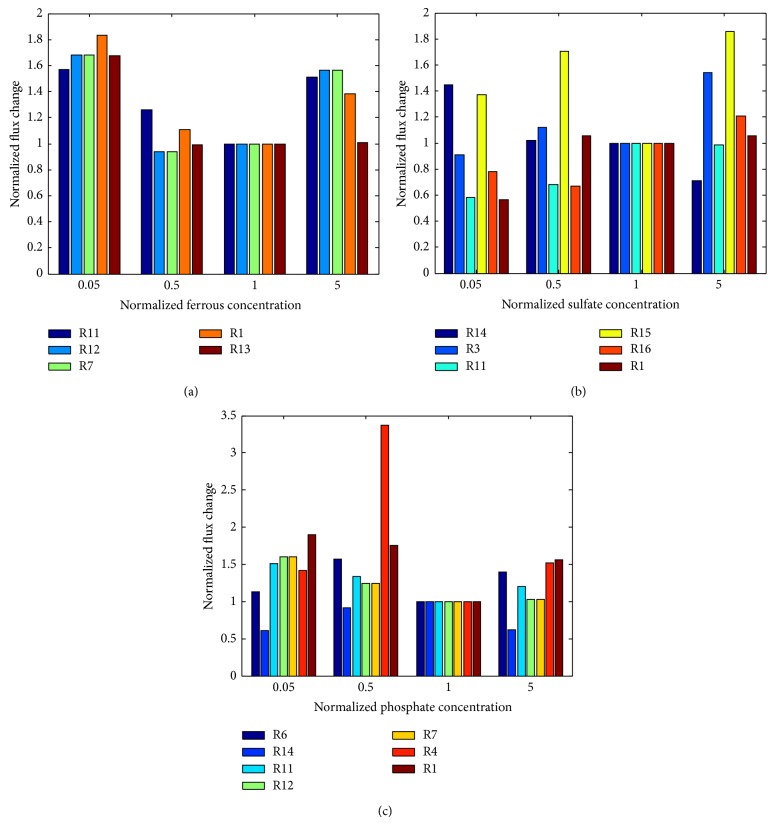
The relative flux change for the biofilm-associated reactions with large flux change upon the change of ferrous ion concentrations (a), sulfate concentrations (b), and phosphate concentrations (c).

In order to further investigate the mechanisms used by* P. aeruginosa* to facilitate its biofilm formation under different availability of phosphate, sulfate, and ferrous ions, the change in the fluxes of the 39 biofilm-associated reactions was evaluated for each of the three ions. Compared to amino acids, the change in the availability of ions does not lead to much change in biofilm formation. This can be reflected in relatively small change in the fluxes through those biofilm-associated reactions. For each of the three ions, the metabolic reactions whose fluxes are of more than 20% change in at least one of the four ion concentrations are identified. The flux change of each of these metabolic reactions is shown in Figure 7, while the metabolic reactions are listed in Table 1 or Table 3. Since the flux change is not that large, the flux of each of these metabolic reactions for each iron concentration is normalized by its value for the reference ion concentration for a better illustration.

Upon the change in the availability of ferrous ion, Figure 7(a) shows that three of the metabolic reactions with large flux change (i.e., Rxn 1, Rxn 7, and Rxn 12) are involved in the arginine metabolism, while the other two reactions are related to acetate metabolism (i.e., Rxn 11) and the production of carbon dioxide (i.e., Rxn 13). Consistent with the biofilm formation profile shown in Figure 4(b), the selected five metabolic reactions have the largest flux change for the low iron concentration. The fluxes decrease and then increase when increasing iron to the medium, high, and very high concentrations. The reactions with large flux change upon the change in the sulfate availability (Figure 7(b)) are mainly involved in magnesium transport (Rxn 14), ammonia production (Rxn 3), tyrosine metabolism (Rxn 11), TCA cycle (Rxn 15 and Rxn 16), and arginine metabolism (Rxn 1). The overestimated biofilm formation for the low sulfate concentrations (shown in Figure 5(b)) is mainly due to the large increase in the fluxes through the exchange reactions for magnesium and citrate, which means that a low sulfate concentration in the medium promotes the exchange of magnesium and citrate which in turn facilitate the biofilm formation. As shown in Figure 7(c), the predicted increased biofilm formation for very low and high phosphate concentrations, which are the major discrepancies between the model prediction and experimental data shown in Figure 6(b), was mainly due to the large flux increase through the biofilm-associated reactions that were involved in the glutamate production (Rxn 6), acetate metabolism (Rxn 11), and arginine metabolism (Rxn 1 and Rxn 4). The enzymes that catalyze these metabolic reactions may need additional regulation for the very low or high phosphate concentration. Further investigation should be focused on the identification of phosphate-regulating signal transduction pathways that regulate these metabolic reactions.

## 3. Discussion

Biofilms are often associated with human diseases, as they can protect pathogens from antibiotics. In addition, it is difficult to eliminate pathogens once they form a biofilm. This work presents the first systemic approach to quantify the ability of* P. aeruginosa* to form a biofilm upon varying the availability of amino acids, iron, sulfate, and phosphate in the medium, using both modeling and experimental approaches. Existing experimental data for* P. aeruginosa* biofilm formation upon the addition of amino acids confirmed the model predicted results for the impact of amino acids on* P. aeruginosa* biofilm formation. The modeling approach was then used to further predict* P. aeruginosa* biofilm formation for the change of ferrous ions, sulfate, and phosphate concentrations in a large range. Experiment was performed to validate those model predictions. Our experimental data by themselves are of value to the biofilm research community as they quantify* P. aeruginosa* biofilm formation for both very low and high concentrations of ions, which were seldom investigated in those existing experimental data. Our modeling approach can predict the trend of* P. aeruginosa* biofilm formation for all four concentrations of ferrous ions and sulfate. Although our approach overestimates the biofilm formation for the low phosphate concentration, it still correctly captures the change of biofilm formation for the low and medium concentrations. In addition to qualitatively predict the trend of the biofilm formation, 75% of the model-predicted biofilm formation value falls within or close to the error bar of the experimental value. The major discrepancies between model prediction and experimental data are found in the underestimation of biofilm formation for the starved ferrous ion concentration (i.e., 0.05-fold) and the overestimation for the starved and rich phosphate concentrations (i.e., 0.05- and 5-fold). The biofilm formation is a complicated process in which the bacteria regulate their metabolism upon the surrounding environmental conditions by down- or upregulating the enzymes that catalyze certain metabolic reactions. This work is mainly based upon a metabolic model, which generally consists of metabolic reactions constrained by expression levels of metabolic genes or activation levels of enzymes. These genes or enzymes are regulated by biochemical reactions used by* P. aeruginosa* to sense the change in the environmental condition and then regulate intracellular enzymes. These biochemical reactions, which are even not completely known, construct signaling pathways, a different type of reaction networks from metabolic networks. In order to improve the model prediction for extreme conditions that are characterized by starved and rich ion concentrations, further research needs to conduct to identify all the signaling proteins involved in these signaling pathways, estimate all kinetic constants in the signaling pathway, and then integrate these pathways with the metabolic model for the ion starved and rich conditions. Nevertheless, the developed modeling approach provided very good prediction performance for all ions in their low and medium concentrations.

The flux change through the 39 biofilm-associated reactions was quantified for all concentrations in this work. Based on this, the reactions with large flux change upon the change of the availability of amino acids, iron, sulfate, and phosphate were identified. It was found that reactions involved in arginine metabolism have large flux changes upon the change of the availability of the aforementioned nutrient components. In addition to arginine metabolism, reactions for ammonia production, the conversion of 4-maleylacetoacetate to 4-fumarylacetoacetate, and the magnesium transport reaction were of large flux change upon the change of the availability of several nutrient components studied in this work. These reactions indicate how* P. aeruginosa* changes its metabolism for the biofilm formation in response to the change in the nutrient availability. Some of these reactions or metabolisms were implied by existing experimental data for their important role in regulating* P. aeruginosa* biofilm formation. For example, enhanced availability of arginine is reported to promote* P. aeruginosa* biofilm formation [16].

*Pseudomonas aeruginosa* was selected as a model species for the biofilm formation in this work because it is one of the most extensively studied gram-negative bacteria that cause chronic wound infections. While the developed approach was applied to study* Pseudomonas aeruginosa* biofilm formation, it can be generalized to other biofilm-associated pathogens. Specially, for each target pathogen, gene expression data obtained for the biofilm mode can be used to identify reactions that are positively associated with the biofilm formation. With the substitution of the biofilm-associated reactions and the metabolic model of* Pseudomonas aeruginosa* with those of the target pathogen, the developed approach can quantify the biofilm formation of the target pathogen upon the change in the availability of environmental nutrients.

Although the influence of a single nutrient component on* P. aeruginosa* biofilm formation was investigated in this work, the framework developed here can be used as a platform to study the impact of the combination of multiple nutrient components. Specifically, the uptake rates for multiple nutrient components can be changed simultaneously to obtain the flux change curve over all biofilm-associated reactions, which can in turn be used to quantify biofilm formation capability upon the change of the availability of multiple nutrient components. Although this is an interesting topic to study and of value for guiding experimental design, this work mainly focused on changing the availability of a single nutrient component in standard media, as this is what most of current experimental research is focusing on.

## 4. Materials and Methods

### 4.1. Experimental Design to Quantify the Biofilm Formation of* P. aeruginosa* upon Differential Availabilities of Ferrous Ions, Sulfate, and Phosphate

All biofilm experiments were conducted at 37°C using* P. aeruginosa* PA14. LB medium was used for growing cells, and M9 medium supplemented with 0.4% glucose was used for performing the biofilm assays. The concentrations of sulfate, ferrous ion, and phosphate in M9-glu medium (named as reference concentrations in the following text) are characterized by 1 mM MgSO_4_, 0.01 mM Fe (NH_4_)_2_(SO_4_)_2_, 49.3 mM Na_2_HPO_4_, and 22 mM KH_2_PO_4_, respectively. Overnight cultures from two independent colonies were diluted to an optical density at 600 nm of 0.05 in M9-glu medium containing various amounts (i.e., 0.05, 0.5, 1 and 5 times the reference concentration) of sulfate, ferrous, and phosphate concentration. The cell suspensions were then transferred to 96-well polystyrene plates, and the plates were incubated at 37°C for 24 h without shaking. Biofilm formation was measured by performing crystal violet assay as described earlier (refer to Lee et al., 2009 [26], for the detail). Total biofilm biomass (absorbance at OD_540_) was normalized by total cell mass (absorbance at OD_600_) to obtain values for normalized biofilm formation and each data point was averaged from 10 replicate wells (five wells per independent culture).

### 4.2. The Flux Balance Analysis (FBA)

**Figure 8:**
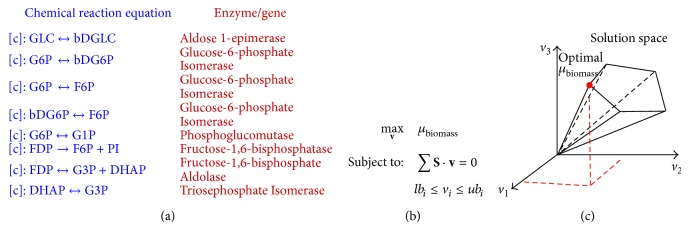
Schematic representation of the flux balance analysis approach on studying the microorganism metabolism. (a) A small fraction of the metabolic reactions taken from the metabolic model, (b) the mathematical representation of the metabolic reaction networks where** S** is the stoichiometric matrix, *μ*_biomass_ is the biomass growth rate,** v** is the flux vector, and *lb*_*i*_ and *ub*_*i*_ are the lower and upper bounds of flux *v*_*i*_, and (c) the optimal growth rate *μ*_biomass_ and metabolite production rates *v*_*i*_, *i* = 1, 2, and 3, determined from the solution space via the flux balance analysis. Three fluxes are used here for the purpose of illustration.

Flux balance analysis is one of the most commonly used approaches to quantify microbial growth for specific nutrient conditions. The metabolic network developed by Oberhardt et al., 2010 [15], is used in this work. It consists of 1056 genes, 883 reactions, and 760 metabolites.  Figure 8(a) lists a portion of metabolic reactions from the model, which is mathematically represented by the stoichiometric matrix** S** in Figure 8(b). The nutrient condition is specified by the lower and upper bounds (i.e., *lb*_*i*_ and *ub*_*i*_ in Figure 8(b)) of the exchange reactions for nutrient components. Large upper bounds are assigned for those nutrient components that are abundant in the surrounding environment. It is assumed in the flux balance analysis that bacteria try to grow as fast as possible from the available nutrients. Therefore, the biomass growth rate *μ*_biomass_ is maximized upon the constraints imposed by the mass balance and the lower/upper bounds of fluxes. This turns to a linear optimization problem where the maximal *μ*_biomass_ is determined from the feasible solution space (Figure 8(c)). The COBRA toolbox developed by Dr. Palsson's group at UCSD is used in this work to perform the flux balance analysis [17].

### 4.3. The Approach to Quantify the Ability of Planktonic* P. aeruginosa* to Form Biofilms for the Nutrient Conditions with Different Availabilities of Amino Acids and Ions (i.e., Phosphate, Sulfate, and Ferrous)

The similarity in the shape and magnitude of the flux change curve (Figure 1(c)) were used by our previous approach [9] to cluster single mutants into different groups to identify the gene targets for eliminating planktonic* P. aeruginosa* before it forms a biofilm. However, no approach has been proposed to directly quantify biofilm formation capability of planktonic* P. aeruginosa* from the flux change curve, in which the flux changes of different biofilm-associated reactions are quite different. To address this, we will define a new criterion from the flux change curve to quantify the ability of* P. aeruginosa* to form a biofilm. In addition, we will formulate an approach to solve a problem that has not been systemically investigated, that is, how to quantify the influence of the availability of environmental nutrients (including amino acids and ions such as phosphate, sulfate, and ferrous ions) on* P. aeruginosa* biofilm formation. Our approach consists of the following steps.

**Figure 9:**
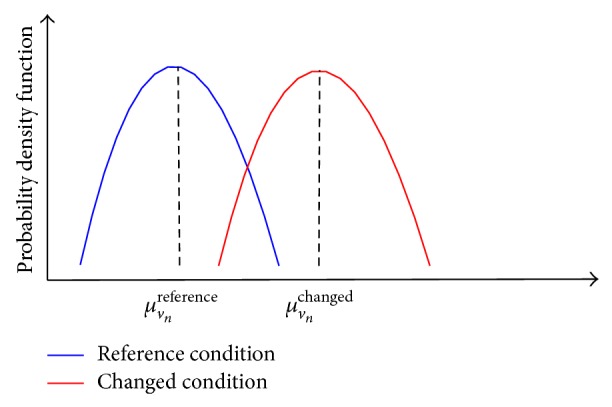
The distributions of fluxes via the *n*th biofilm reaction for both the reference and changed nutrient conditions. Both the probability density function and the mean value are determined for each distribution.

(i) Define the reference and changed nutrient conditions for changing the availability of amino acids, phosphate, and sulfate and in the medium.
 In the amino acid study, only one amino acid is added at a time to the medium to mimic a changed nutrient condition with different amino acid availability. The maximal uptake rate in FBA for each amino acid is determined from the literature (i.e., Oberhardt et al., 2008 [7]). Four different concentrations are studied for each of the three ions. The maximal uptake rate for each ion is determined by fitting the biomass growth rate to the experimental data, while the biofilm formation is then predicted from the model. In order to study the influence of phosphate, sulfate, and ferrous ions on biofilm formation, a large range of availability concentrations are investigated for each ion in order to capture the complete picture on how the starved and rich availabilities of these ions affect biofilm formation and the biomass growth rate of* P. aeruginosa*.
(ii) Sample the fluxes of each biofilm-associated reaction for both the reference and changed nutrient conditions using the ACHR sampling approach from the COBRA toolbox [17].
 As shown in Figure 9, the mean value and the probability density function of the sampled fluxes are calculated for the reference nutrient condition (i.e., *μ*_*v*_*n*__^reference^ and *f*_*v*_*n*__^reference^) and for the changed nutrient condition (i.e., *μ*_*v*_*n*__^changed^ and *f*_*v*_*n*__^changed^).
 The change in the flux distribution of each biofilm-associated reaction upon the change of the nutrient condition, that is, Flux_Var_*v*_*n*__^nutrient *m*^, is quantified by
  (1) $$\begin{array}{c} {\text{Flux}\_\text{Var}}_{v_{n}}^{\text{nutrient}\;m}=\frac{\mu_{v_{n}}^{\text{changed}}}{\mu_{v_{n}}^{\text{reference}}}\times \text{KS}\left(f_{v_{n}}^{\text{changed}},f_{v_{n}}^{\text{reference}}\right), \end{array}$$
 where KS(·) is a two-sample Kolmogorov-Smirnov test in which the test result is equal to one/zero if the distribution *f*_*v*_*n*__^changed^ is statistically/not different from *f*_*v*_*n*__^reference^.
(iii) Use (2) to quantify biofilm formation in the defined nutrient condition:
  (2) Cbiofilmnutrient m=∑i=1nFlux_Varvinutrient m2×sign(Flux_Varvinutrient m)n,
 where the sign(·) function is equal to one if the flux through the biofilm-associated reaction *v*_*i*_ does not change its direction upon the change of the availability of nutrient component *m*; that is, Flux_Var_*v*_*i*__^nutrient *m*^ is positive. The rationale behind this is that reaction *v*_*i*_ is positively associated with biofilm formation and a changed flux direction means the trend to form biofilms reverses. *C*_biofilm_^nutrient *m*^ indicates biofilm formation capability of* P. aeruginosa* upon the change of the availability of nutrient component *m*. A value of *C*_biofilm_^nutrient *m*^ that is significantly larger than one means that* P. aeruginosa* increase its biofilm formation once the availability of nutrient component *m* is changed from the reference to the changed condition.

## Figures and Tables

**Table 1:** Top ten biofilm-associated reactions with large flux increase upon addition of amino acids into the medium (adapted from [9]).

Reactions	Biological subsystems
Rxn 1: 2 H^+^ + H_2_O + urea → CO_2_ + 2 ammonium	Arginine metabolism
Rxn 2: acetate + ATP + coenzyme A → acetyl-CoA + AMP + diphosphate	Pyruvate metabolism
Rxn 3: L-threonine → 2-oxobutanoate + ammonium	Ammonia production
Rxn 4: 2 ATP + L-glutamine + H_2_O + bicarbonate → 2 ADP + carbamoyl phosphate + L-glutamate + 2 H^+^ + phosphate	Arginine metabolism
Rxn 5: nicotinamide adenine dinucleotide + O-phospho-4-hydroxy-L-threonine → 2-amino-3-oxo-4-phosphonooxybutyrate + H^+^ + nicotinamide adenine dinucleotide – reduced	Pyridoxine metabolism
Rxn 6: 4-aminobutanoate + 2-oxoglutarate → L-glutamate + succinic semialdehyde	Glutamate production
Rxn 7: L-aspartate + ATP + L-citrulline → AMP + N(omega)-(L-arginino)succinate + H^+^ + diphosphate	Arginine metabolism
Rxn 8: reduced glutathione + methylglyoxal → (R)-S-lactoylglutathione	Pyruvate metabolism
Rxn 9: S-adenosyl-L-methionine + butyryl-[acyl-carrier protein] → 5-methylthioadenosine + acyl carrier protein + H^+^ + N-butyryl-L-homoserine lactone	Quorum sensing
Rxn 10: alpha-oxobenzeneacetic acid *↔* benzaldehyde + CO_2_	CO_2 _production

**Table 2:** Reference concentrations for each ion in experiment.

Ions	Carrier formula	Reference concentration
Ferrous ion	Fe(NH_4_)_2_(SO_4_)_2_	5 × 10^−4^ mM
Sulfate	MgSO_4_	0.05 mM
Phosphate	Na_2_HPO_4_ and KH_2_PO_4_	4.93 mM Na_2_HPO_4_ and 2.2 mM KH_2_PO_4_

**Table 3:** Biofilm-associated reactions with large flux change upon the change of ferrous ions concentrations (adapted from [9]).

Reactions	Biological subsystems
R 11: 4-maleylacetoacetate → 4-fumarylacetoacetate	Acetate metabolism
R 12: N(omega)-(L-arginino)succinate *↔* L-arginine + fumarate	Arginine metabolism
R 13: ATP + oxaloacetate → ADP + CO_2_ + phosphoenolpyruvate	Carbon dioxide production
R 14: mg2[e] → mg2[c]	Magnesium transport
R 15: citrate[e] + H^+^[e] *↔* citrate[c] + H^+^[c]	TCA cycle
R 16: ATP + coenzyme A + succinate *↔* ADP + phosphate + succinyl-CoA	TCA cycle
